# Kisspeptin and GPR54 immunoreactivity in a cohort of 518 patients defines favourable prognosis and clear cell subtype in ovarian carcinoma

**DOI:** 10.1186/1741-7015-5-33

**Published:** 2007-11-15

**Authors:** Leah M Prentice, Christian Klausen, Steve Kalloger, Martin Köbel, Steven McKinney, Jennifer L Santos, Challayne Kenney, Erika Mehl, C Blake Gilks, Peter Leung, Ken Swenerton, David G Huntsman, Samuel AJ Aparicio

**Affiliations:** 1 Molecular Oncology and Breast Cancer Program, British Columbia Cancer Research Centre and Department of Pathology, University of British Columbia, Vancouver, British Columbia, Canada; 2 Genetic Pathology Evaluation Centre of the Prostate Centre and Departments of Pathology of Vancouver Coastal Health Research Institute, British Columbia Cancer Agency, and University of British Columbia, Vancouver, British Columbia, Canada; 3 Department of Obstetrics and Gynaecology, University of British Columbia, Vancouver, British Columbia, Canada; 4 Institute of Pathology, Charité Hospital, Berlin, Germany; 5 Department of Gynaecology, Vancouver General Hospital, Vancouver, British Columbia, Canada; 6 Cheryl Brown Ovarian Cancer Outcomes Unit, Department of Gynaecologic Oncology, British Columbia Cancer Agency, Vancouver, British Columbia, Canada; 7 Department of Medicine, University of British Columbia, Vancouver, British Columbia, Canada; 8 Department of Medical Oncology, British Columbia Cancer Agency, Vancouver, British Columbia, Canada

## Abstract

**Background:**

Kisspeptins and their G-protein coupled receptor, GPR54 are required for GnRH release and have been associated with anti-metastatic tumour cell behaviour in model systems. The latter might suggest that their overexpression would be associated with a better prognosis in cancer. However, kisspeptin/GPR54 interactions (autocrine, paracrine, and/or endocrine) could also impact tumour behaviour in a negative manner. Here, for the first time, we associate the immunoreactivity of the kisspeptin/GPR54 ligand-receptor pair with favourable prognosis in a large cohort of ovarian carcinomas.

**Methods:**

Immunohistochemical analysis for kisspeptin and GPR54 was performed on a tissue microarray (TMA) consisting of 518 early stage ovarian carcinomas, all with linked clinical outcome data. The TMA was scored using a staining intensity scale of 0 (negative), +1 (mild-moderate), and +2 (strong). Strong staining cases were considered either kisspeptin or GPR54 positive and designated as 1, while all other cases were considered negative and designated 0. All statistical analysis was conducted using two-sided tests and a p value equal to or less than 0.05 was considered significant.

**Results:**

Kisspeptin and GPR54 immunoreactive cases show a favourable prognosis in univariable disease specific survival (p = 0.0023, p = 0.0092), as well as in overall survival (p = 0.0006, p = 0.0002). Furthermore, kisspeptin is an independent marker for favourable prognosis as determined by multivariable disease specific (p = 0.0046) and overall survival analysis (p = 0.0170), while GPR54 is an independent marker for overall survival only (p = 0.0303). Both kisspeptin positive and GPR54 positive cases are strongly associated with the ovarian carcinoma clear cell subtype (p < 0.0001, p < 0.0001), and GPR54 is significantly associated with favourable prognosis in overall survival within the clear cell subtype (p = 0.0102).

**Conclusion:**

Kisspeptin and GPR54 immunoreactivity are significantly associated with favourable prognosis in both disease specific and overall survival, as well as being significantly associated with the clear cell ovarian carcinoma subtype, thereby creating the first independent prognostic biomarkers specific for ovarian clear cell carcinomas.

## Background

The early diagnosis and management of ovarian cancer is a major area of unmet medical need. Central to the lack of progress in clinical management has been the virtual absence of prognostic or predictive molecular markers for ovarian cancer. Key to addressing these questions is the availability of sufficiently large, clinically annotated tissue microarrays (TMA) that offer the prospect of defining the prognostic or predictive value of any given molecular marker. Therefore we have constructed a large ovarian cancer TMA (518 patients) with associated clinical demographic and outcome information and have used this to systematically address the value of possible biomarkers of disease prognosis. In the present study, we have tested the prognostic value of kisspeptin and GPR54 immunoreactivity in ovarian cancer. Kisspeptins (Kp-54, Kp-14, Kp-13, Kp-10) are the canonical, physiologically occurring and high affinity RF-amide peptide ligands that activate transmembrane signalling via a classical (7TM1) family G-protein coupled receptor, GPR54. Kisspeptins were first discovered through microcell-mediated chromosome transfer experiments that defined the KiSS-1 locus as a suppressor of melanoma tumour metastasis [1,2]. Subsequently, kisspeptins were associated as endogenous ligands for the GPR54 receptor. Furthermore, a physiological role in the regulation of placental trophoblast invasion has been suggested [3] and in migratory cell lines, activation of GPR54 signalling abrogates migratory behaviour [1,4-6]. Specifically overexpression of KiSS-1 in an ovarian cell line expressing endogenous GPR54 suppressed its metastatic phenotype [7].

In 2003, we uncovered in human and mouse genetic studies, the major physiological functions of kisspeptin-GPR54 signalling, as being gatekeepers for GnRH release in the hypothalamus [8,9]. In the absence of functional kisspeptin [10] and GPR54 [9,11-13] neither humans nor mice undergo puberty and are unable to generate pituitary release of gonadotropins that drive sex-steroid release. Several subsequent physiological studies have confirmed that kisspeptins act as neuroendocrine peptides that switch on or off the GnRH axis in humans and mammals [14-25], and are thus required as physiological regulators of sex-steroid release. The mechanistic relationship between GPR54 regulation of the hypothalamic-pituitary-gonadal axis, and possible effects on epithelial cell migration remains unclear, however several anecdotal studies on human tumours have suggested possible associations of loss/absence of expression, with poor prognosis [26-33]. Recently, Zhang et al [34] and Hata et al [35] surveyed RNA expression of the KiSS-1 and GPR54 loci in small cohorts (< 100 cases) of ovarian cancer and observe a trend towards favourable prognosis where KiSS-1/GPR54 RNA expression is elevated. None of these studies have been sufficiently powered to address cell type and prognostic associations in major epithelial malignancies. We show in the present study of 518 ovarian cancer cases that kisspeptin and GPR54 immunoreactivity are very significantly associated with a clear cell carcinoma subtype, and that both kisspeptins and GPR54 are independent markers for favourable prognosis as determined by multivariable analysis.

## Methods

### Ovarian tumour samples and TMA construction

Approval for the study was obtained from the ethics committee of the University of British Columbia. Most women diagnosed with ovarian cancer in British Columbia are treated at the British Columbia Cancer agency (BCCA) and provincial treatment guidelines are followed. Outcomes are tracked via The Cheryl Brown Ovarian Cancer Outcomes Unit as an ovarian cancer database of the BCCA. A total of 3501 patients with invasive epithelial ovarian carcinoma were referred to the BCCA between 1984 and the year 2000. The focus of this study was 834 patients who had ovarian carcinoma with no macroscopic residual disease after surgery. For 202 cases, the slides of the primary ovarian tumour were not available for review and these cases are excluded. A gynaecological pathologist (CBG) then did a blinded full slide review of the remaining 632 cases. Tumour cell type and grade (Silverberg) were assessed; all clear cell carcinomas were considered to be grade 3, as per World Health Organization recommendations. After review, 518 cases of invasive ovarian carcinoma were available in tissue blocks for tissue microarray construction. A representative area of each tumour was selected and a duplicate core TMA was constructed (Beecher Instruments, Silver Springs, MD, USA); the cohort is described in Table 1. Serial 4 μm sections were cut for immunohistochemical (IHC) analysis.

**Table 1:** Clinicopathological characteristics of the cohort

**Parameter**	**N**
**Histopathological subtype**	
Adenocarcinoma	4
Clear cell	132
Endometrioid	125
Mucinous	31
Serous	212
Squamous cell	1
Transitional	6
Undifferentiated	7
**Grade**	
1	106
2	114
3	298
**Stage**	
1	214
2	219
3	85
**Age (years)**	
Mean (SD)	58 (12.8)
Median	57
Range (min-max)	25–89

### Immunohistochemistry

#### Kisspeptin

**Figure 1:**
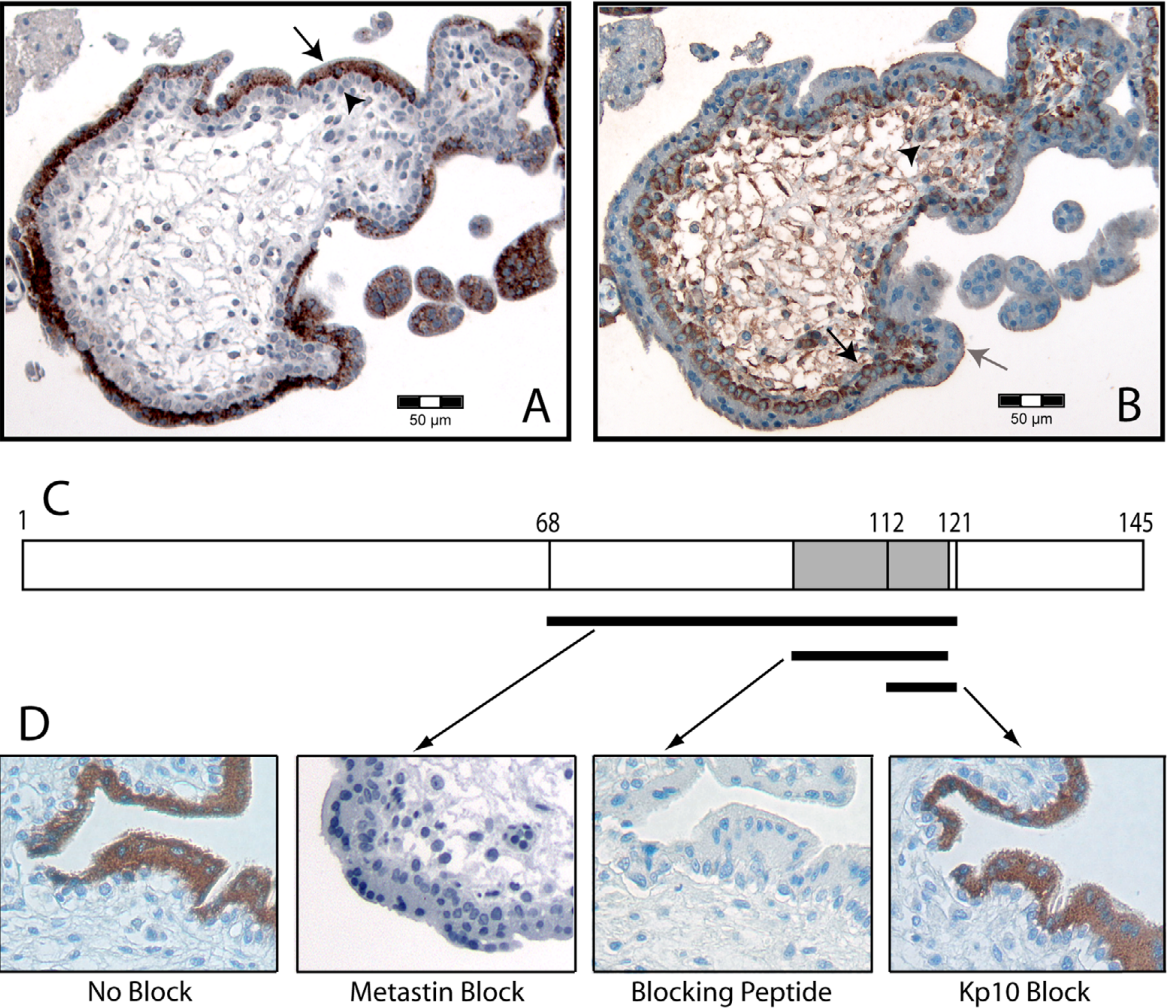
**IHC controls**. Less than 10-week-old human placenta used as a positive control. (A) Kisspeptin-IR shows intense cell-type specific staining in the syncytiotrophoblasts (black arrow), while the cytotrophoblast layers remain unaffected (black arrowhead). (B) GPR54-IR shows intense staining in the villous cytotrophoblasts (black arrow), the extravillous cytotrophoblasts (black arrowhead), and moderate staining on the syncytiotrophoblast membrane (grey arrow). (C) Schematic of the 1–145 amino acid (aa) KiSS-1 pro-peptide. Metastin (Kp-54) is encoded within the 68–121 aa sequence, while Kp-10 is encoded within this same region from 112–121 aa. The specific blocking peptide is encoded within the 100–120 aa sequence. (D) Varying kisspeptin-IR was found among the different blocking peptides used. Blocking the primary antibody with full-length metastin (Kp-54) and blocking peptide resulted in complete loss of immunoreactivity, while Kp-10 was unable to block any detectable staining.

Sections from formalin-fixed and paraffin-embedded tissues were deparaffinized with xylene and rehydrated with a graded series of alcohols. Wet heat-induced antigen retrieval was performed in a steamer for 20 min with a modified citrate buffer (pH 6.1, Dako, Mississauga, Ontario, Canada). Following antigen retrieval, sections were treated with 3% hydrogen peroxide (H_2_O_2_) in phosphate buffered saline (PBS) for 30 min to quench endogenous peroxidase activity. All of the aforementioned steps were followed by three washes with PBS for 5 min each. Slides were subsequently blocked for 30 min with serum-free protein block (Dako) and incubated overnight at 4°C with a polyclonal goat anti-(KiSS-1) antibody (C-20, Santa Cruz Biotechnology, Santa Cruz, CA, USA) diluted 1:400 in serum-free protein block. Kisspeptin immunoreactivity (IR) was detected with the CSA II biotin-free tyramide signal amplification system and 3,3'-diaminobenzidine chromogen solution (Dako). Specifically, rabbit anti-goat horseradish peroxidase conjugate (HRP) was applied for 15 min followed by fluorescyl-tyramide amplification reagent for 15 min and anti-fluorescein HRP for 15 min. All of the steps subsequent to the incubation with primary antibody were followed by three washes with Tris-buffered saline containing 1% Tween (TBST) for 5 min each. Slides were counterstained with Harris hematoxylin (Sigma-Aldrich, Oakville, Ontario, Canada) and mounted in a xylene-based mounting medium. Based on previously published data showing cell-type restriction of GPR54 and kisspeptins in different trophoblast layers of human placenta [3], less than 10-week old human placenta was used as a specificity control (courtesy of Vancouver Coastal Health archives), in conjunction with two blocking peptides (21 residues and 54 residues; Figure 1). Omission of the primary antibody was used as a negative control.

#### GPR54

TMA 4 μm sections were processed using a Ventana Discovery XT automated system (Ventana Medical Systems, Tucson, AZ, USA) as per manufacturer's protocol with proprietary reagents. After slides were baked at 60°C for 1 h, they were deparaffinized on the automated system with EZ Prep solution (Ventana). Heat-induced antigen retrieval method was used in Cell Conditioning solution (CC1-Tris based EDTA buffer, pH 8.0, Ventana). The polyclonal rabbit GPR54 antibody was obtained from MBL International Corporation (Woburn, MA, USA) specific for the N-terminal extracellular domain (catalogue number LS-A1929) and used with heat at a 1:25 concentration in Ventana antibody diluent. The Ventana Universal Secondary Antibody was used for 32 min at 37°C. The detection system used was the Ventana DABMap kit, and slides were then counterstained with Hematoxylin and treated with a proprietary bluing agent (Ventana). All washes were conducted with the Ventana Reaction Buffer. Dehydration steps and coverslip procedure were completed manually as per manufacturer's recommendations. Specificity was determined by Western blot (Additional file 1) and by using less than 10-week old human placenta as a positive control (Figure 1) and omission of primary antibody as a negative control.

#### Photomicrographs

The TMA was digitally scanned with a BLISS (Bacus Laboratories Inc., Slide Scanner) automated system (Bacus Laboratories, Lombard, IL, USA) as previously described [36]. These images are available on our webslide server that is publicly available [37].

### Statistical analysis

Survival time dependant recursive partitioning was used to binarise the raw kisspeptin and GPR54 data. Univariable survival analysis was performed by the generation of Kaplan-Meier curves [38] and differences between the groups were assessed using Log-rank Statistic [39]. Multivariable survival analysis was performed using the Cox Proportional Hazards Model [39,40]; the adenocarcinoma, squamous cell, transitional, and undifferentiated ovarian subtypes were excluded from multivariable analysis due to insufficient sample size. Contingency tables and the Pearson Chi-square statistic were used to test the change in the distribution of kisspeptin and GPR54 expression across primary cell types [41]. All analyses were performed using JMP version 6.0.3 (SAS Institute, Cary NC, USA).

## Results

### Kisspeptin positivity is an independent marker for favourable prognosis

Kisspeptin-IR was tested on human placenta less than 10 weeks old as a positive control (Figure 1). There was cell type specificity demonstrated by intense staining in syncytiotrophoblast cells as previously determined [3,42], but not in other cell layers of the trophoblast. Pre-absorption with two different blocking peptides (metastin (Kp-54, 68–121 amino acids (aa)) and kisspeptin 100–120 aa), fully blocked kisspeptin-IR, whereas Kp-10 (112–121 aa) showed little or no block (Figure 1).

**Figure 2:**
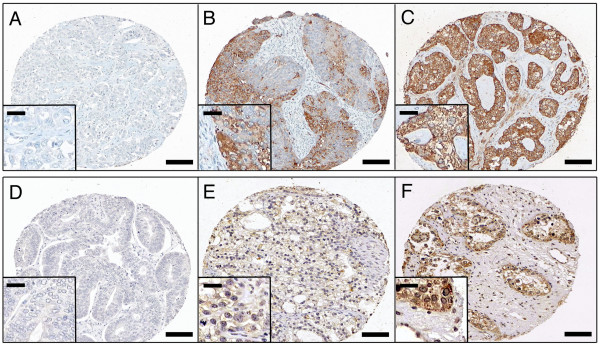
**Immunoreactivity patterns**. Three types of kisspeptin-IR (A-C) and GPR54-IR (D-F) observed in the ovarian TMA. (A, D) Representative samples of negative cases show complete lack of staining and are classified as 0. (B) Moderate GPR54-IR shows a patchy staining pattern with light and dark brown regions of reactivity shown throughout the tumour core, and (E) Mild kisspeptin-IR shows uniform light brown staining throughout the sample: both B and E are classified as +1 immunoreactivity. (C, F) Examples of +2 intense immunoreactivity exhibit dark brown staining in all tumour cells. Scale bar represents 100 μm. Insets in each panel show a more detailed view of the staining pattern that is demonstrated in the larger image; inset scale bar represents 25 μm.

**Figure 3:**
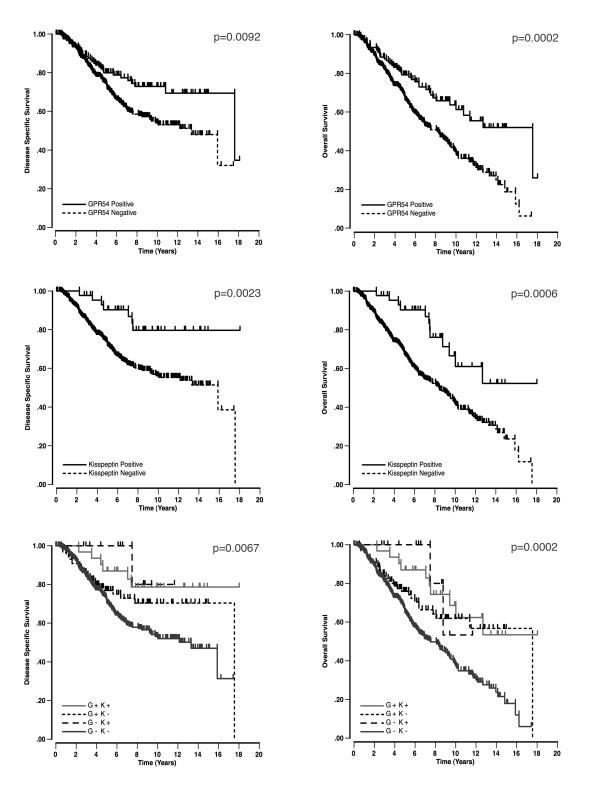
**Disease specific (left) and overall (right) survival curves for kisspeptin and GPR54**. The top two graphs demonstrate the significant survival curves for GPR54, while the middle two graphs demonstrate kisspeptin related survival. For the bottom two graphs, the p value refers to the distance between the GPR54 positive/kisspeptin positive cases (G+ K+, solid light grey) and the GPR54 negative/kisspeptin negative cases (G- K-, solid dark grey).

For the 518 case ovarian tissue microarray, kisspeptin-IR was scored as 0 for negative cases, +1 for mild staining, and +2 for intense staining (Figure 2). Of the 518 cases, 44 stained at +2, 98 had +1 staining intensity, 354 cases were negative for kisspeptin-IR, and 22 cases were uninterpretable. The negative (0) and mildly reactive (+1) cases were grouped for statistical analysis and assigned the designation 0 and considered kisspeptin negative, while the +2 cases were considered kisspeptin positive and designated as 1. Univariable disease specific survival analysis showed that kisspeptin-IR significantly associated with favourable prognosis (p = 0.0023), as did overall survival (p = 0.0006, Figure 3). Further, multivariable survival analysis including; stage, grade, histological subtype, age and GPR54-IR, indicated kisspeptin-IR as an independent marker for favourable prognosis in disease specific (p = 0.0046, Table 2) and overall survival (p = 0.0170, Table 3).

**Table 2:** Multivariable disease specific proportional hazards

**Parameter**	**Risk ratio (95% CI)**	**p Value**
Stage		< 0.0001
1	0.6404 (0.4901 to 0.8278)	
2	0.7149 (0.5647 to 0.8994)	
3	1.0000	
Histological grade		0.0720
1	0.6234 (0.3829 to 0.9808)	
2	1.3899 (1.0193 to 1.8888)	
3	1.0000	
Subtype		0.2508
Clear cell	1.4519 (0.8853 to 2.3847)	
Endometrioid	0.6804 (0.4105 to 1.1032)	
Mucinous	1.0721 (0.5119 to 1.9699)	
Serous	1.0000	
Age		0.0747
	N/A	
GPR54		0.1118
Positive	0.6475 (0.3738 to 1.1052)	
Negative	1.0000	
Kisspeptin		0.0046
Positive	0.3508 (0.1426 to 0.7408)	
Negative	1.0000	

*RR for age is not available because it is a continuous variable.

**Table 3:** Multivariable overall proportional hazards

**Parameter**	**Risk ratio (95% CI)**	**p Value**
Stage		< 0.0001
1	0.7258 (0.5842 to 0.8961)	
2	0.7149 (0.6457 to 0.9476)	
3	1.0000	
Histological grade		0.5356
1	0.8268 (0.5818 to 1.1642)	
2	1.1113 (0.8647 to 1.4148)	
3	1.0000	
Subtype		0.6763
Clear cell	1.2126 (0.7934 to 1.8513)	
Endometrioid	0.8120 (0.5508 to 1.1914)	
Mucinous	1.0192 (0.5652 to 1.6841)	
Serous	1.0000	
Age		< 0.0001
	N/A	
GPR54		0.0303
Positive	0.5959 (0.3684 to 0.9523)	
Negative	1.0000	
Kisspeptin		0.0170
Positive	0.4844 (0.2443 to 0.8841)	
Negative	1.0000	

*RR for age is not available because it is a continuous variable.

### GPR54 positivity is an independent marker for favourable prognosis in overall survival

The GPR54 antibody and protocol were tested on less than 10-week old human placenta and specifically stained both villous and extravillous cytotrophoblasts and the syncytiotrophoblasts as described by previous groups [3,42] (Figure 1). Although GPR54 is a 7-transmembrane protein, there was some reactivity in the cytoplasm of some tumour cells (this is not entirely surprising as GPR54 is a transmembrane protein and can be recycled through the cytoplasm) but only membranous staining was taken into consideration while assessing immunoreactivity. Three immunoreactivity patterns were observed within the TMA for GPR54. Specifically, negative or very weak reactivity in less than 5% of cells was designated as 0 (103 cases), while patchy or moderate staining in 5–75% of cells was designated +1 (282 cases), and strong staining in greater than 75% of cells were considered +2 (104 cases, Figure 2). The remaining nine cases were uninterpretable. As with kisspeptin, the 0 and +1 GPR54 cases were group together and considered as loss of receptor and designated 0, while the strong staining +2 cases were considered positive and designated as 1. Univariable survival analysis determined GPR54 as a significant marker for favourable prognosis in disease specific (p = 0.0092) and overall survival (p = 0.0002, Figure 3). Similar to kisspeptin-IR, GPR54 maintained significance in multivariable overall survival (p = 0.0303, Table 3). However, GPR54 was not found to be a significant independent marker in disease specific survival (p = 0.1118, Table 2).

### Kisspeptin positivity correlates with GPR54 positive cases

Kisspeptin positive cases had a moderate correlation with GPR54 positivity as determined by Kendall's tau-b [43] (τ = 0.3837, p < 0.0001). There were 31 cases that were both kisspeptin and GPR54 positive, 90 cases that were kisspeptin negative and GPR54 positive, 12 cases with kisspeptin positivity and had loss of GPR54, 356 cases that had loss of both kisspeptin and GPR54, and the remaining 29 cases were uninterpretable. When kisspeptin-IR and GPR54-IR cases are grouped together (G+ K+), patients have a more favourable outcome than those that have loss of either one or both (G- K+, G+ K-, G- K-). There is a significant difference between survival for double positive patients (G+ K+) as compared to double negative patients (G- K-) in both disease specific (p = 0.0067) and overall survival (p = 0.0002, Figure 3).

### Kisspeptin and GPR54 positive staining are significantly associated with clear cell carcinoma histopathological subtype

The percentage of kisspeptin and GPR54 positive cases within each histopathological subtype is listed in Table 4. The proportionality of primary histopathological cell type in the entire cohort, kisspeptin positive cases, and GPR54 positive cases are represented in Table 5. Testing for an association between ovarian carcinoma subtype and kisspeptin status, there was a highly significant positive association with clear cell carcinoma, and a significant negative association with serous carcinoma subtype (χ^2^, p < 0.0001). GPR54 positive cases also had a significant positive association with clear cell carcinoma subtype and a negative association with the serous subtype (χ^2^, p < 0.0001).

**Table 4:** Percentage of kisspeptin and GPR54 positive cases within the histological subtypes

**Histological subtype**	**Kisspeptin positive (%)**	**GPR54 positive (%)**
Clear cell	21.88	66.41
Endometrioid	8.13	20.33
Mucinous	12.00	10.34
Serous	1.49	3.37

**Table 5:** Kisspeptin and GPR54 proportions within the cohort

**Histological subtype**	**Whole cohort**		**Kisspeptin positive**		**GPR54 positive**	
	
	**Proportion**	**Count**	**Proportion**	**Count**	**Proportion**	**Count**
Clear cell	0.2640	132	*0.6364	28	**0.7131	87
Endometrioid	0.2500	125	0.2273	10	0.2049	25
Mucinous	0.0620	31	0.0682	3	0.0246	3
Serous	0.4240	212	*0.0682	3	**0.0574	7

*χ^2 ^p value < 0.0001

**χ^2 ^p value < 0.0001

**Figure 4:**
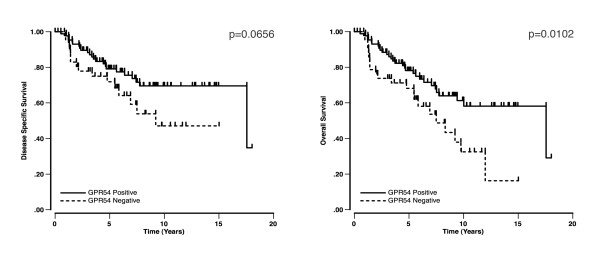
Kaplan-Meier disease specific (left) and overall (right) survival curves for GPR54 positivity within the clear cell subtype.

When disease specific survival and overall survival were analyzed within each ovarian carcinoma subtype, the log-rank test for kisspeptin-IR status failed to achieve significance (due to insufficient sample size), although for the clear cell cases statistical significance was approached (p = 0.1042, p = 0.0859, results not shown). Of note, none of the kisspeptin positive patients that were not clear cell subtype (16 cases) died from their disease. Similarly, when assessing GPR54 positivity within each ovarian cancer subtype, disease specific survival did not reach significance within the clear cell subtype (p = 0.0656), although significance was achieved for overall survival (p = 0.0102, Figure 4).

## Discussion

Although clear cell carcinomas comprise fewer than 5% of ovarian malignancies, they are notoriously difficult to treat due to their tendency to resist platinum based chemotherapy [44]. To date, clinical stage has been the only prognostic marker for clear cell ovarian carcinoma. Here we show for the first time, that kisspeptin and GPR54 immunoreactivities mark distinctly for favourable prognosis, with kisspeptin being independent of pathologic subtype, stage, grade, or age in both overall and disease specific survival, while GPR54 is an independent marker in overall survival. Within clear cell carcinomas, GPR54 expressers have a favourable prognosis and to our knowledge this is the first molecular marker of prognosis specifically applicable to clear cell ovarian cancer. Although several studies have suggested possible relationships between GPR54 and kisspeptin expression and clinical outcome [26,27,30-34,45], these studies have consisted of smaller cohorts and while some associations have been noted, some studies might not have been sufficiently powered to address possible prognostic or cell type specific effects with rigour. To date, the present study is the largest systematic analysis of GPR54 and kisspeptin expression determined by immunoreactivity for an epithelial malignancy. In part, this could be due to difficulties in obtaining sufficiently specific antisera and detection protocols, in that short peptides and GPCRs are notoriously difficult antibody targets. The antibodies and immunodetection protocols used in this study were verified by the use of either Western blotting and cell-type specific expression (GPR54), or cell-type specific expression and specific blocking peptides (kisspeptin). This is based on previous work showing differential expression of kisspeptin and GPR54 in human placental trophoblast cell types [3,45]. We note that while Muir et al [46] demonstrated a 75 kDa fragment as GPR54 by Western blot, our data show a fragment much closer to the predicted 42.5 kDa molecular weight for GPR54. The basis of this difference is unknown, but could result from post-translational modification arising in different tissues (brain vs tumour cell lines). Although blocking peptides suggest specificity of the antisera used, the precise spectrum of immunoreactivity of the kisspeptin antiserum to kisspeptin fragments remains to be determined. As with many antibodies, it remains possible that other proteins could be detected however this does not diminish their utility as markers of prognosis. Very recently a survey of 76 ovarian cancer patients using Q-PCR detection of GPR54 and kisspeptin transcripts [35] demonstrated a negative correlation between KiSS-1 and GPR54 mRNA levels with residual disease, although they showed no correlation with histopathological subtype (possibly due to the relatively small number of clear cell ovarian cancers in that cohort), however the overall correlation observed in this study is in agreement with our observations.

The mechanisms responsible for the association of kisspeptin and GPR54 expression with disease behaviour in ovarian cancer requires definitive studies, however several possibilities arise. It is possible that expression of kisspeptins and/or GPR54 result in higher endogenous GPR54 signalling in malignant cells. Although no studies have directly addressed the degree of GPR54 signalling in epithelial malignancies in relation to clinical outcomes, the present study shows that both kisspeptin and GPR54 expression are associated with a better prognosis. Furthermore, patients with double positive tumours (G+ K+) have the most favourable prognosis (Figure 3). These observations together with previous evidence of the effects of GPR54 signalling on cell migration, suggest some form of autocrine or paracrine loop could exist in clear cell carcinomas. GPR54 is exquisitely sensitive to kisspeptin ligand stimulation [3,5] and receptor overexpression alone might be enough to increase basal signalling through GPR54.

The interplay of mechanisms could be complicated by the major physiological role of GPR54, which is to regulate GnRH secretion at the hypothalamic level. Kisspeptins can cross from the peripheral circulation to act on the hypothalamus, as has been shown in numerous mammalian [22,24,47-49] and one human study [21]. It is possible that kisspeptin overexpressing tumours could result in stimulation of the hypothalamic-pituitary axis, resulting in the release of gonadotropins and other derived peptides with a possible paracrine/endocrine effect on tumour growth. Indeed Nash et al [50], have shown that melanoma cells unable to signal on exposure to kisspeptins, can still be suppressed from metastasis by exogenous kisspeptin, suggesting that paracrine effects could operate in these cases. Finally, some evidence suggests that kisspeptins and GPR54, which are expressed in ovarian epithelium and granulosa cells, might co-modulate the activity of gonadotropins in sex steroid release [42]. If such a mechanism were operational in clear cell ovarian cancers, it would imply that tumour behaviour is also linked to co-modulatory peptides.

Beyond the salient observation of prognostic significance in this study, the nature of the proteins involved suggests a number of possible areas for intervention. First, kisspeptins, the products of the KiSS-1 gene locus, are naturally occurring peptides that can be detected in human serum and other tissues [51-53]. It is possible that serum kisspeptide levels could be developed as a biomarker of disease activity in patients with clear cell carcinoma. However, diagnostic grade antibodies would have to be developed before routine immunohistochemical-based analysis of kisspeptin and GPR54 could be undertaken. Secondly, kisspeptins are naturally occurring peptide hormones that have activity in humans [21]. As such they are highly amenable to use as therapeutic agents, either alone or as modified peptides. We anticipate that the strong association of GPR54 and kisspeptin expression with outcome and clear cell type in ovarian carcinoma will stimulate fresh approaches to what is still a lethally intractable disease.

## Conclusion

Kisspeptin and GPR54 are significantly associated with favourable prognosis in both disease specific and overall survival, as well as being significantly associated with the clear cell ovarian carcinoma subtype, thereby creating the first independent prognostic biomarkers specific for ovarian clear cell carcinomas.

## Competing interests

The author(s) declare that they have no competing interests.

## Authors' contributions

LMP was responsible for the IHC data accruement and analysis, participated in the study design, wrote the initial manuscript, and implemented the manuscript revisions. CK optimized the Kisspeptin IHC, acquired the data and assisted in the study design.

SK was responsible for the statistical analysis, and assisted with the editing of the manuscript. MK was responsible for the IHC analysis and assisted with the editing of the manuscript. SM assisted with the data analysis and editing of the manuscript.

KS and JLS led the ovarian cancer cohort design, data accruement for the cohort, and assisted with editing of the manuscript. CK and EM were responsible for GPR54 IHC optimization and staining. CBG assisted in the study design, data accruement, data analysis and editing of the manuscript. PL assisted with the study design. DGH and SAJA led the study design, writing and editing of the manuscript.

All authors have read and approved the final manuscript.

## Pre-publication history

The pre-publication history for this paper can be accessed here:



## Supplementary Material

Additional file 1 **Western blot demonstrating GPR54 specificity**. A total of 30 μg of protein was run on 12% SDS-PAGE and transferred to a nitrocellulose membrane. The membrane was blocked for 1 h at room temperature with TBST 5% non-fat milk powder and incubated overnight at 4°C on a rocking incubator with 1/1000 GPR54 MBL antibody. The blot was washed four times with TBST for 5 min each and incubated with a 1/20000 secondary anti-rabbit antibody for 1 h at room temperature. The blot was then incubated with SuperSignal Chemiluminescent (Pierce, San Francisco, CA, USA) for 5 min and exposed to film for 20 s before developing. Loading control β-actin was detected using 1/2500 anti-(β-actin) antibody incubated on the same blot for 1 h at room temperature and visualized with an anti-mouse secondary antibody and enhanced chemiluminescence (ECL) for a 12 min exposure. The cell lines were kept in tissue culture and passaged as per distributors' recommendations. Protein lysate was collected using the standard RIPA buffer method. Click here for file
