# Supplementary figures and images for: Kisspeptin and GPR54 immunoreactivity in a cohort of 518 patients defines favourable prognosis and clear cell subtype in ovarian carcinoma
Source: BMC Med. 2007 Nov 15;5:33. doi: 10.1186/1741-7015-5-33 (PMC2200658; doi:10.1186/1741-7015-5-33)

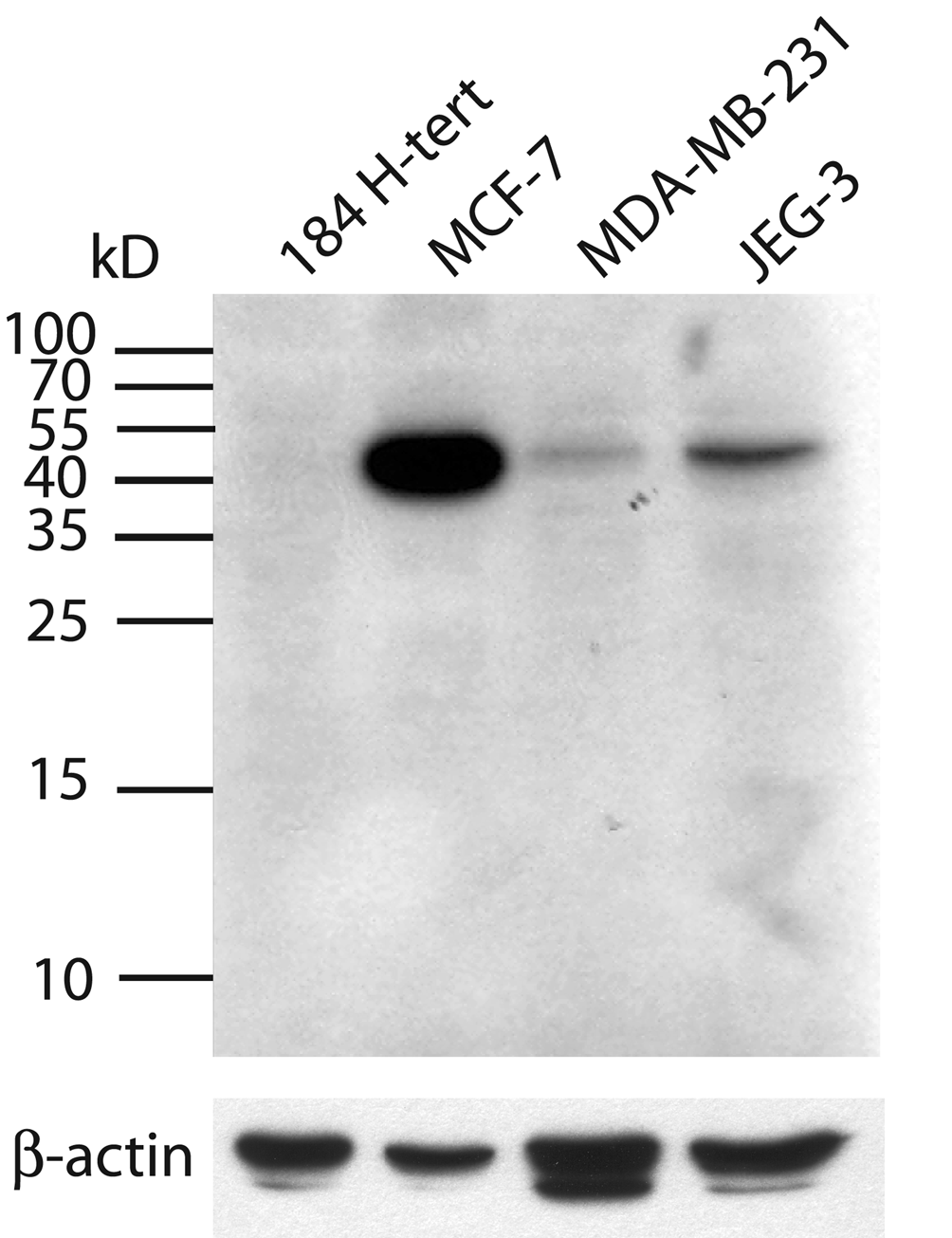

Supplement: Additional file 1 — Western blot demonstrating GPR54 specificity. A total of 30 μg of protein was run on 12% SDS-PAGE and transferred to a nitrocellulose membrane. The membrane was blocked for 1 h at room temperature with TBST 5% non-fat milk powder and incubated overnight at 4°C on a rocking incubator with 1/1000 GPR54 MBL antibody. The blot was washed four times with TBST for 5 min each and incubated with a 1/20000 secondary anti-rabbit antibody for 1 h at room temperature. The blot was then incubated with SuperSignal Chemiluminescent (Pierce, San Francisco, CA, USA) for 5 min and exposed to film for 20 s before developing. Loading control β-actin was detected using 1/2500 anti-(β-actin) antibody incubated on the same blot for 1 h at room temperature and visualized with an anti-mouse secondary antibody and enhanced chemiluminescence (ECL) for a 12 min exposure. The cell lines were kept in tissue culture and passaged as per distributors' recommendations. Protein lysate was collected using the standard RIPA buffer method. [file 1741-7015-5-33-S1.tiff]
